# How to plan reintroductions of long-lived birds

**DOI:** 10.1371/journal.pone.0174186

**Published:** 2017-04-26

**Authors:** Virginia Morandini, Miguel Ferrer

**Affiliations:** Applied Ecology Group, Estación Biológica de Doñana, CSIC, Seville, Spain; Sichuan University, CHINA

## Abstract

Reintroductions have been increasingly used for species restoration and it seems that this conservation tool is going to be more used in the future. Nevertheless, there is not a clear consensus about the better procedure for that, consequently a better knowledge of how to optimize this kind of management is needed. Here we examined the dynamics of released long-lived bird populations (lesser kestrel, *Falco naumanni*, Bonelli's eagle *Aquila fasciata*, and bearded vulture *Gypaetus barbatus*) in object-oriented simulated reintroduction programs. To do that, number of young per year and number of years of released necessary to achieve a successful reintroduced population were calculated. We define a successful reintroduction as one in which when the probability of extinction during two times the maximum live-span period for the species (20, 50, and 64 years respectively) was less than 0.001 (P<0.001) and they showed a positive trend in population size (r>0.00). Results showed that a similar total number of young (mean 98.33±5.26) must be released in all the species in all the scenarios in order to get a successful reintroduction. Consequently, as more young per year are released the new population is going to be larger at the end of the simulations, the lesser the negative effects in the donor population and the lowest the total budget needed will be.

## Introduction

The global loss of biodiversity is a well-documented phenomenon, with increasing numbers of species at risk of extinction due to direct or indirect anthropogenic causes, e.g. [1, 2]. Management to reduce risk of species extinction includes a wide variety of actions, one of the more intensive of which is reintroduction. Reintroductions are intentional translocations of species into parts of their historically known range from which they have been extirpated [3]. Wildlife reintroductions are becoming increasingly common, being now considered to be an important tool for conservation of endangered or threatened species [4, 5].

In an attempt to improve success in reintroductions projects, the International Union for the Conservation of Nature (IUCN) Guidelines for Re-introductions were published in 2013 providing specific policy guidelines for each phase of a reintroduction project [3]. Also, other authors suggest a series of standards for documenting and monitoring the methods and outcomes associated with reintroduction projects for birds [2, 6].

The final objective of any reintroduction is the persistence of the new population without intervention, but it is not very clear what criteria would be used to define what constitutes a successful reintroduction [6–12]. Some definitions of success that have been proposed include: (i) breeding by the first wild-born generation [13]; (ii) a three-year breeding population with recruitment exceeding adult death rate [13]; (iii) an unsupported wild population of at least 500 individuals [14]; or (iv) the establishment of a self-sustaining population [8, 15]. However, a major problem with defining a reintroduction as a success or a failure is that, by any criteria, this definition is limited in time [7]. Even taking this into account still we need some objective criteria to decide when to stop releasing animals. This is important in order to plan any reintroduction adequately as well as to get the necessary political and public support for a long term conservation action [16, 17].

One of the first challenges for a reintroduction is to secure the source of animals to be released. There are two main sustainable sources of animals for a reintroduction program: extraction from wild populations or breeding in captivity. After an intensive human persecution during the second half of the 20th century, many endangered species, such as raptors, persist at high local density in small and relatively isolated populations [18]. This is a common pattern for most of large endangered species, which have suffered in the past from habitat destruction and human persecution. Those remaining high-density small populations of raptors often show low fecundity, resulting from density-dependent reproduction [19–21]. Because fecundity is low, public opinion is not very prone to extractions, making sensible management difficult, especially if extractions can put the donor population at risk [22]. Consequently, it is necessary to analyze the potential effect that different strategies of repeated extractions would have on donor population.

Another important consideration for reintroductions is the monetary expense. However, this factor is often overlooked when planning reintroductions [22]. Especially when animals are sourced from captive propagation programs, reintroductions may be an expensive option for managers of endangered species [22]. On the other hand, young released per year determines the length of the reintroduction project, affecting in a very significant way the total cost of these programs [22].

In this paper, we present different simulated scenarios of extractions and releases of animals, identify the impact of each scenario on monetary cost of reintroduction programs and discuss a criterion to define success in reintroductions. We present the results for simulated reintroduction programs of long-lived birds, selecting three different species that vary in body size, fecundity and population dynamics: the small lesser kestrel, *Falco naumanni*, the medium-size Bonelli´s eagle *Aquila fasciata*, and the large bearded vulture *Gypaetus barbatus*.

We examine the potential success of reintroductions under our different simulated scenarios, combining number of young per year and the length of the reintroduction necessary for each species with the effect of the repeat extraction of young in the donor population, in an attempt to find an optimal combination of monetary cost and probability of success. We try to determine the minimum number of young that we need to release each year and the minimum number of years of releases for each species as well as the final population size of the new population in each one of the scenarios

## Material and methods

### The species

The lesser kestrel (*Falco naumanni)* is a small (120–145 g), long-lived (maximum live-span 10 years) colonial falcon, being females large than males [23] and references therein. The species feeds mainly on invertebrates, and has experienced a marked decline in some areas of its breeding range during the last 30 years, being the target species for several reintroduction programs [24]. The lesser kestrel data on demographic parameters was taken from literature [23] coming from a color-ringing and monitoring of breeding performances in 12 colonies in the Seville province (Spain) during 6-year period (1988–1993).

The Bonelli’s eagle (*Aquila fasciata*) is a Mediterranean and long-lived bird of prey of medium size (1,600–2,200 g) with a maximum life span of 25 years [25]. The species has experienced a severe decrease in Spain during the past decades, mainly as a result of power line accidents and human persecution [26]. Currently the species appears to be recovering slowly but its conservation status is under discussion [27]. Bonelli’s was extirpated from the Balearic Island (Spain) during the 20th century and a reintroduction program to recover this population started in 2011. Estimates for Bonelli`s eagle were taken from literature [25] and were based on data of 7 subpopulations in Spain summarizing 142 pairs that were surveyed during 1994–2005 period.

The bearded vulture (*Gypaetus barbatus*) is a large (4,500–7,000 g) long-lived territorial raptor, with a maximum life span of 32 years [22] and references therein, that breeds mainly in mountainous areas [28]. The species feeds mainly on bones and meat of ungulates which it swallows whole or in pieces. During the 20th century its numbers and distribution area declined due to human persecution and at present, three reintroduction programs are running in Europe, one in Switzerland and two in Spain [22]. Estimates of demographic parameters for bearded vultures were taken from literature [22, 28]. Data was from the only Spanish population of the species situated in Pyrenees. The population increases from 40 pairs in 1970 to 150 by 2011.

### Simulations

We conducted simulations to analyze the viability of reintroduction programs for this three species under different scenarios. We used the Vortex simulation software (Vortex, version 10.0.76, [29, 30]). Vortex is an individual-based model for population viability analyses (PVA). It models population dynamics as discrete, sequential events that occur according to probabilities defined by the user, and model constant or random variables that follow specified distributions. The events used for modeling describe the typical life cycle of sexually reproducing, diploid organisms. This method is particularly appropriate for species showing low fecundity, long life span, small population size, estimable age-specific fecundity and survival rates, and monogamous breeding, as in the species and populations we modeled here [31]. In fact, Vortex has already been used to analyze the viability of populations of Bonelli´s eagles [25] or bearded vultures [22]. In the bearded vulture study, reintroduction scenarios and effect in donor populations were analyzed. Using estimates of fecundity and mortality rates for the three species previously published (Table 1) we conducted several simulations for different scenarios, performing 1000 replicates for each one.

**Table 1:** Summary of parameter values used in Vortex for the simulations of trends in the donor population and in the hypothetical reintroduced population.

Parameter	Lesser kestrel	Bonelli’s eagle	Bearded vulture
Age of first breeding	2	4	7
Maximum live-span of reproduction	10	25	34
Maximum number of broods per year	1	1	1
Maximum progeny per brood	4	2	1
Sex ratio at birth	50%	50%	50%
Density dependent fecundity rate	1.99 at low density 1.50 at high density	1.02 at low density 0.78 at high density	0.60 at low density 0.35 at high density
Preadult mortality	64%	73%	50%
Adult annual mortality	20%	8.53%	6%

Based on data from [23], [25] and references therein and [22] and references therein.

First, we simulated reintroduction programs of the three species considered. To do that, we calculated the number of released juveniles per year and during how many years that we need to achieve a successful new population. We considered a new population to be successful when the probability of extinction during two times the life-span period for the species (lesser kestrel: 20 years, Bonelli`s eagle: 50 years and bearded vulture: 64 years) was less than 0.001 (*p*<0.001) and they showed a positive trend in population size (r>0.00). We simulated reintroduction programs from 5 to 20 years of duration calculating the minimum number of juveniles we have to release per year with a sex ratio of 1:1. We consider the minimum number of young in each scenario the values below which the probability of extinction of the simulated population was >0.001. In these simulations, no density-dependence or ceiling population limit was considered due to small size of the simulated population at the end of the simulations.

Second, we simulated for each one of the species and scenarios, the effect on the wild donor populations of repeated extraction of the minimum number of young needed for a successful reintroduction according previous simulations. In order to standardize the potential effect of repeat extractions on wild donor populations among species, we simulated for each one of the three species a donor population of the necessary size to produce 100 young per year. According to the mean fecundity for each species (see Table 1), we need 26 pairs (100 individuals of all ages) of lesser kestrels to produce 100 young per year, 40 pairs of Bonelli’s eagles (140 individuals in total) and 83 pairs of bearded vultures (335 individuals). We set donor populations at their maximum limit when the simulation started and we introduced density dependence in fecundity in the simulations as shown in Table 1. Simulations started with a stable age distribution and equilibrate sex ratio (1:1).

### Cost analyses

We estimated the annual cost of a standard reintroduction program based on extraction of young from wild populations, using data from the following reintroduction programs developed in Spain: (i) osprey reintroductions in Huelva and Cádiz [32]; (ii) the Spanish imperial eagle reintroduction in Cádiz [33] and (iii) the bearded vulture reintroduction in Cazorla (http://www.gypaetus.org/) and in Picos de Europa (Asturias, Spain, http://www.quebrantahuesos.org/). The estimate cost includes the personal necessary to take care of the extracted young until de age of release, the feed and monitoring during the dependence period until the young leaves the area, plus the cost of emitters, hacking towers and educational programs. Obviously, the costs could change through time, but it is the relative costs of the different strategies that are important here.

### Statistical analyses

We tested for trends with linear analysis and we used the F-ratio statistic to test the slope. Variances of the linear models were tested for homogeneity using Cochran’s C statistic. GLM with appropriate distribution and link function were used to assess differences among species or scenarios. Friedman ANOVA tests were used to examine differences in mean number of breeding pairs in reintroduced or donor populations according different strategies of extraction and releases. Statistical significance was set at *p*<0.05 and analyses were conducted using the STATISTICA 8 package (Statsoft Inc., Tulsa, OK, USA).

## Results

**Fig 1:**
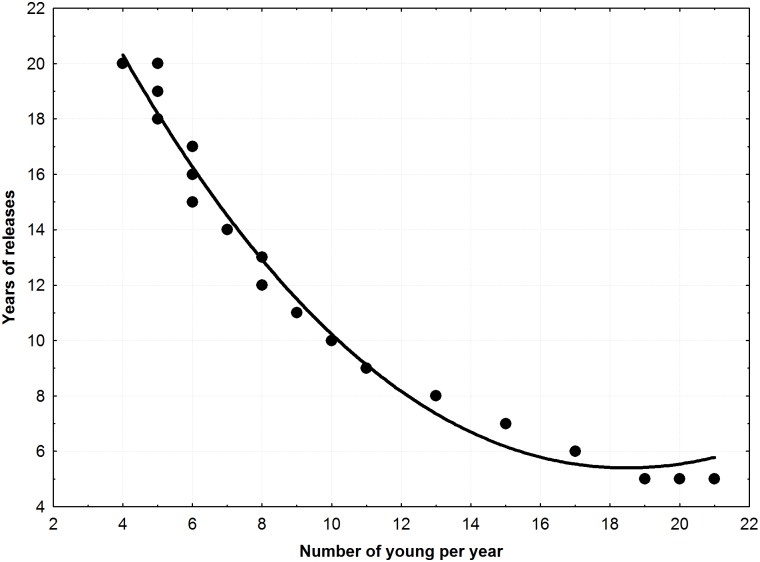
Negative exponential relationship (*r* = -0.934, n = 48, *p*<0.001) between the number of young released per year and number of years necessary to obtain a probability of extinction below 0.001 for all the species.

Different combinations of number of released young each year and number of years of releases to obtain a successful reintroduction (Probability of extinction < 0.001 and r > 0) are show in Table 2. For all the species, the minimum number of young per year necessary for a successful reintroduction varies from 18 young per year during 5 years to 5 young per year during 20 years. Interesting, in all the cases and with all the species, a similar total number of young (mean 98.33±5.26) must be released in order to get a successful reintroduction. No significant effect of the species in the number of young to release was found, being only significant the number of years of releases (GLM normal distribution and log link function, “years”: Wald statistic = 1402.05, *p*<0.001; “species”: Wald statistic = 0.94, *p* = 0.625). A negative significant exponential relationship between young per year and years of releases was found (r = -0.934, n = 48, *p*<0.001). Consequently, number of years necessary to obtain a successful reintroduction increases exponentially as we decrease the number of young released per year (Fig 1).

**Table 2:** Different combinations of young released per year and number of years of released to achieve successful reintroductions.

Species	Years of releases	Young per year	Total number of young	Stochastic *r*	Sd (*r*)	Probability of extinction	Number of pairs
Lesser kestrel	5	20	100	0.056	0.164	<0.001	19.81
	10	10	100	0.090	0.159	<0.001	16.94
	15	6	90	0.110	0.167	<0.001	13.36
	20	4	80	0.127	0.184	<0.001	9.33
Bonelli´s eagle	5	19	95	0.051	0.153	<0.001	17.91
	10	10	100	0.063	0.164	<0.001	16.83
	15	6	90	0.066	0.161	<0.001	14.19
	20	5	100	0.067	0.158	<0.001	11.43
Bearded vulture	5	21	105	0.022	0.129	<0.001	14.92
	10	10	100	0.032	0.139	<0.001	13.79
	15	6	90	0.038	0.142	<0.001	13.37
	20	5	100	0.042	0.142	<0.001	11.79

Simulation time was double the live-span period for the species (lesser kestrel: 20 years, Bonelli's eagle: 50 years and bearded vulture: 64 years).

Total number of young was years of releases*young per year.

Number of pairs was the mean value of the 1000 replicates performed for each scenario at the end of the simulation.

**Fig 2:**
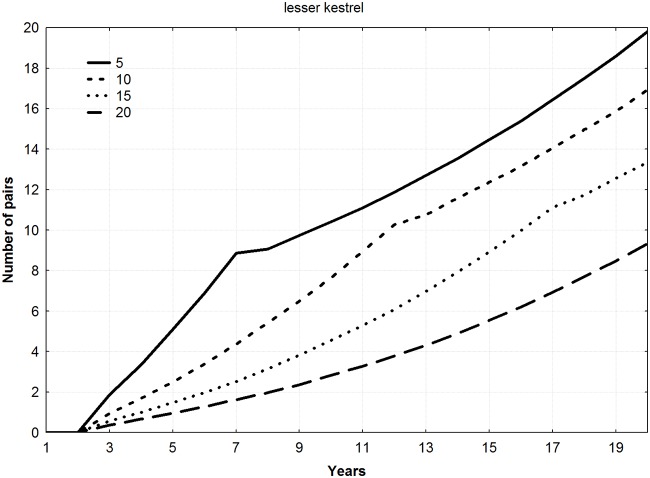
Trajectories of new populations according to different combinations of young released per year and duration of the releases for the specie *Falco naumanni* (5 years–20 young, 10 years–10 young, 15 years–6 young, and 20 years–5 young).

**Fig 3:**
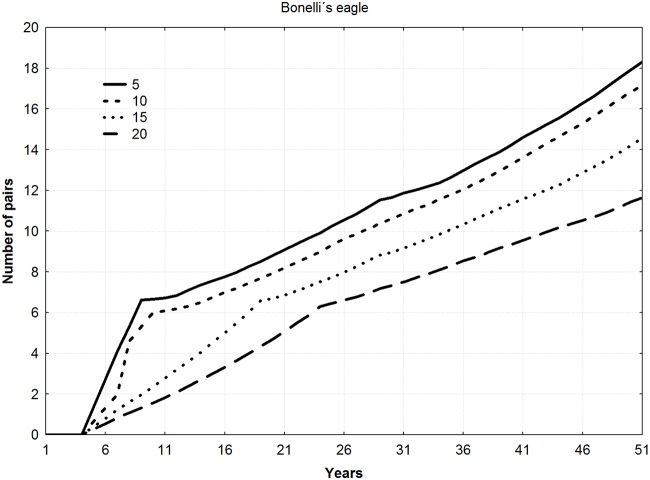
Trajectories of new populations according to different combinations of young released per year and duration of the releases for the specie *Aquila fasciata* (5 years–20 young, 10 years–10 young, 15 years–6 young, and 20 years–5 young).

**Fig 4:**
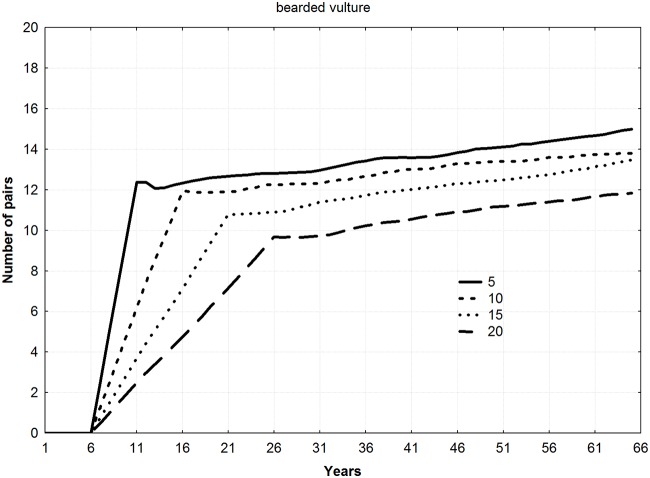
Trajectories of new populations according to different combinations of young released per year and duration of the releases for the specie *Gypaetus barbatus* (5 years–20 young, 10 years–10 young, 15 years–6 young, and 20 years–5 young).

For all the species, final mean population levels increased with the number of young released per year (ANOVA F = 9.22, *p* = 0.011), thereby shortening the duration of the reintroduction (Figs 2–4). No differences among species in final number of pairs were found (ANOVA F = 1.13, *p* = 0.383).

Mean number of pairs in the new populations during all the years of simulation showed a significant relationship with number of young released per year, increasing as number of juvenile individuals released per year increased (Friedman ANOVA; lesser kestrel: chi square = 54, n = 20, df = 3, *p*<0.001; Bonelli´s eagle: chi square = 138, n = 50, df = 3, *p*<0.001; bearded vulture: chi square = 174, n = 64, df = 3, *p*<0.001).

**Fig 5:**
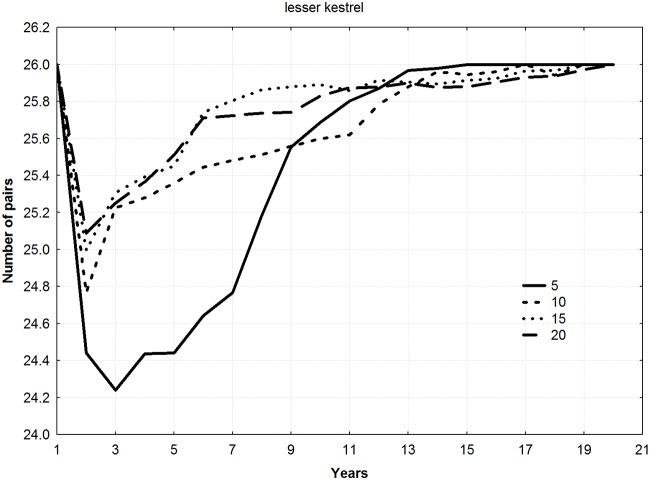
Effect of different combinations of young removed per year and number of years of extraction on the number of breeding pairs in the donor population the specie *Falco naumanni* (5 years-20 young, 10 years-10 young, 15 years-6 young and 20 years-5 young).

**Fig 6:**
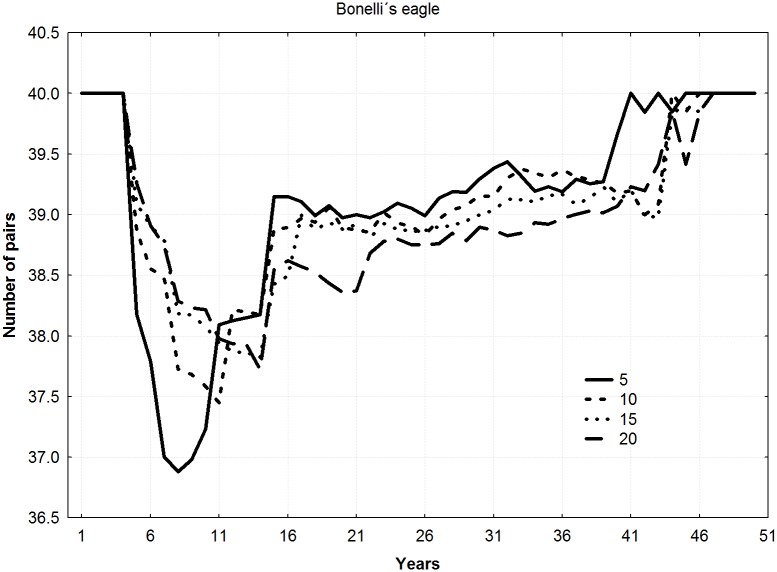
Effect of different combinations of young removed per year and number of years of extraction on the number of breeding pairs in the donor population the specie *Aquila fasciata* (5 years-20 young, 10 years-10 young, 15 years-6 young and 20 years-5 young).

**Fig 7:**
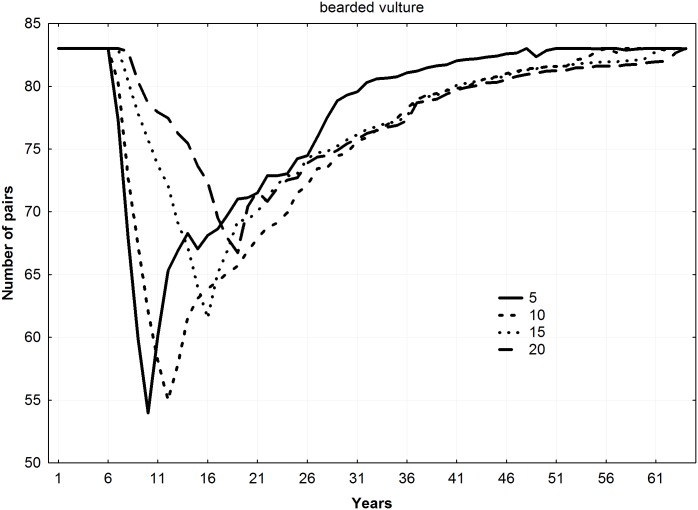
Effect of different combinations of young removed per year and number of years of extraction on the number of breeding pairs in the donor population the specie *Gypaetus barbatus* (5 years-20 young, 10 years-10 young, 15 years-6 young and 20 years-5 young).

Simulating the removal of nestlings from the donor population resulted in significant variation depending on the duration of extraction (Figs 5–7). The longer the extraction period, the lower the size of the modeled donor population during all the years of simulation was (Friedman ANOVA; lesser kestrel: chi square = 10.45, n = 20, df = 3, *p* = 0.035; Bonelli´s eagle: chi square = 38.56, n = 50, df = 3, *p*<0.001; bearded vulture: chi square = 51.56, n = 64, df = 3, *p*<0.001). However, at the end of all these simulations the number of breeding pairs in the donor populations was the same being always the maximum possible for each one of the species (lesser kestrel = 26, Bonelli´s eagle = 40 pairs and bearded vulture = 83 pairs). The temporary decreases in the number of breeding pairs in donor populations lasted longer as years of extraction increaseds (Figs 5–7). Time required to recover the initial donor population size (standardized according total simulation time) was only affected by the number of young extracted per year (GLM normal distribution and log link function, Wald statistic = 190.7, *p*<0.001), being shorter as the number of young per year increases. Recovery time of the initial donor population size was not significantly affected by species (GLM normal distribution and log link function, Wald statistic = 0.4, *p* = 0.803). Anyways, for all the species and in all the scenarios, the probability of extinction of the donor populations was always below 0.001.

The cost of reintroduction programs based on extraction form wild populations analyzed in Spain (Ospreys, Spanish imperial eagles, and Bearded Vultures), including cost of hacking and any associated costs of the program, give an annual estimated budget of 100 000€. Considering that the main component of the total budget per year is personal salaries and that number of persons needed is nearly the same when releasing 5 or 20 young per year, number of years of releases is the main factor affecting monetary cost of these programs. When captive breeding is used as a source of young for reintroduction programs, account must be taken, in such a long-lived species, of the lengthy period in captivity before individuals taken early in their lives start to breed. Additionally, we have to consider all-year running cost of the necessary facility and personal involved plus the cost of the release of young at the field (the only one we have to consider using extraction of young from wild population). In other words, reintroductions using captive breeding would be around 17 times more expensive than the alternative of harvesting wild young [22].

## Discussion

In this study we use a definition of success in reintroductions that is mainly functional. In small populations of endangered species, population viability analyses are the usual way to make predictions and guide decisions about conservation actions. Reintroduced populations should be no different, as by definition, reintroduced populations are small and endangered at the beginning. The main point for us is not to secure forever this new population but to decide when new releases are not necessary with objective criteria. Using simulations and objective criteria, like probability of extinction below 1% and positive trend during twice the live span of the species, we can make predictions about the length of the program and the number of young to release. Predicted trajectories of the simulations can be used to check annually if the evolution of the new population is over, on, or under expectations. Adjusting the simulated period to twice the life span of the species allows us to compare species with different life histories in comparable units of time. Interestingly, no differences among species in number of young to released, final population size or negative effects on donor population were found, being only significant the number of years of releases or extractions.

Other definitions of success like breeding by the first wild-born generation are dependent of the demography of each particular species and don’t give us any idea about viability of the new established population [13]. The same problems arise with “an unsupported wild population of at least 500 individuals” [14] again depending on the demography of the species (500 individuals would means few breeders or a lot of them), or with “a three-year breeding population with recruitment exceeding adult death rate” [13], giving us no information about viability or predicted persistence of the new population [14]. The establishment of a self-sustaining population [15] is similar to our definition of success but here we propose that the time we have to consider for these calculations must be based on the live-span of the specie, allowing us to compare species with a different live history in comparable units of time.

Results showed that a similar total number of young (mean 98.33±5.26) must be released of all the species and in all the cases in order to get a successful reintroduction. As we decrease number young released per year we need to increases exponentially number of years of releases. In the other hand, the number of young per year also affects in a significant way the final reintroduced population size, the effect of extractions in donor population, and the total cost of the project.

As more young per year are released the larger the new population is going to be at the end of the simulations. These differences are due to a different number of breeding pairs in the new population at the early stage of the reintroduction. When we release a large number of young with the same age, as soon as the survivals reach sexual maturity, the number of pairs is going to increase accordantly, increasing the number of new young born in the new population. In the other hand, even releasing the same total number of young, we have to wait longer an increase in young production of the new population if we release few young per year.

Differences in number of young extracted from the donor population per year have significant effects on the size of the donor population. The size of the modeled donor population became lower as the extraction period lengthened and the number of young extracted per year decreased. The duration of temporary decreases in the number of breeding pairs in donor populations was significantly related to the length of the extraction period, even if in all the cases donor population size was at population ceiling at the end of the simulation. Temporal decreases in the number of breeding pairs would generate a negative public perception of this management action.

Result showed that time necessary to achieve a successful reintroduction increases exponentially as we decreases number of young released per year. As total annual budget of a reintroduction program is, to a certain degree, independent of the number of released young, the most important component of the total cost of these programs is their length number of years. In our case, we can make a reintroduction releasing 20 young per year during 5 years (approximate cost 100,000€ per year, total cost 500,000€) or alternatively with 5 young per year during 20 years (total budget 2,000,000€).

It is important to point out that whatever the number of released young will be, there are additional analyses that must be done to assure a successful reintroduction. Following IUCN guidelines we must be sure that causes that provoke extinction in the past are not operating now. In raptors the main historical factor driving local extinctions was human persecution. Nowadays, human attitude have change substantially allowing the recovery of these former populations, but new threats must also be determined and corrected if necessary (power lines, wind farms, poisoning, etc). Also, habitat availability analysis, including density and diversity of preys must be conducted before any releases.

Summarizing, a good general suggestion is to increase as much as possible the number of released young per year, reducing the length of the program, increasing the final size of the new population, avoiding significant effects on donor population and, of course, using the money in an optimal way. Optimal design of reintroduction program for long-live birds is to use a donor population of the appropriate size (this is always cheaper than any breeding in captivity program [22] and to releases 20 young per year during 5 years, independently of the species. Reducing significantly the total cost and limiting in time the conservation program would increases the public support to these reintroduction actions. Of course, theoretically we can conduct a reintroduction within one year if we release the necessary number of nestlings (around 100 according our results). Nevertheless, we did not conducted these simulations because a unique releases would be very dangerous according environmental (or others) stochastic fluctuations. Episodic effect or any unnoticed mortality factor only detected after the first released would be a high risk.

Many endangered species recovery programs could benefit from these suggestions. Reintroduction programmes of various animals have increased greatly during the last decades, and are likely to become more common in the future [33]. The use of population simulations with objective criteria could reduce the costs, increasing at the same time the probability of success. Additionally, having predicted trajectories for the new and donor populations facilitates critical future monitoring of these reintroduction programs to detect and correct any bias in mortality or fecundity that could put species survivorship at risk.
